# Supplementary material for: Polymorphisms in Pfkelch13 domains before and after the introduction of artemisinin-based combination therapy in Southwest Nigeria
Source: PLoS One. 2025 Mar 31;20(3):e0316479. doi: 10.1371/journal.pone.0316479 (PMC11957316; doi:10.1371/journal.pone.0316479)

Samples: 12944
Bases: 1088
Average spacing: 12

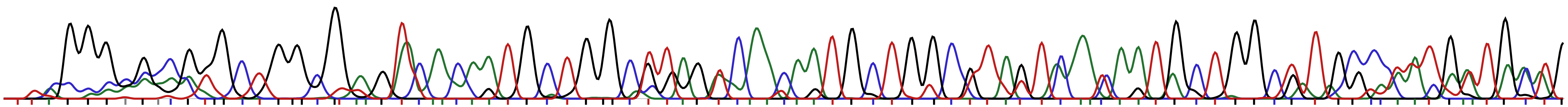

10 20 30 40 50 60 70 80 90
T T A G G G - G - C G TGG C T G GG C G T AA G T C AA C A A T G C T G GG C T T A G T C A A C A A T G C T G G C T T A T T C AAC A A T G C T G G C G T GG CC A A T G AA T G C T G

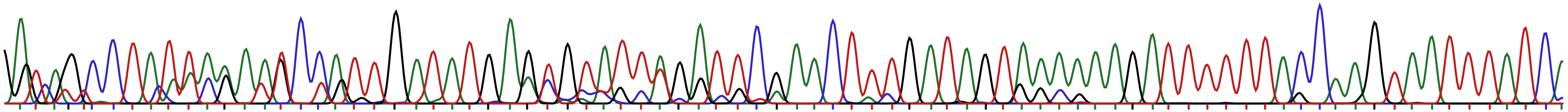

100 110 120 130 140 150 160 170
A T A G T C C T A T T A G A A T C C A T T G A T A T G A G T G T A T T A G A T T C G A A C T T T G A T A G T A A A A A A G A T T T T T T A C C A A G T A A T T T A T C A

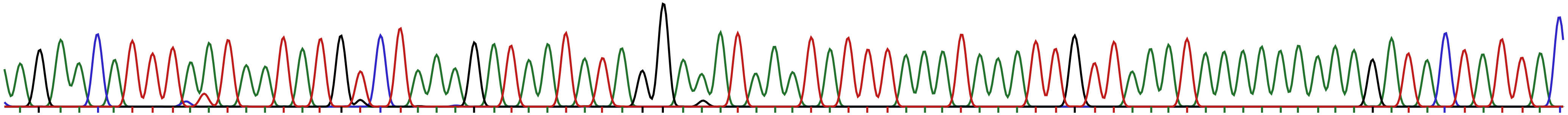

180 190 200 210 220 230 240 250 260
A G A A C A T T T A A T A A T A T G T C T A A A G A T A A T A T A G G A A A T A A A T A T T T A A A T A A A T T G T T A A A T A A A A A A A A A G A T A C T A T T A C

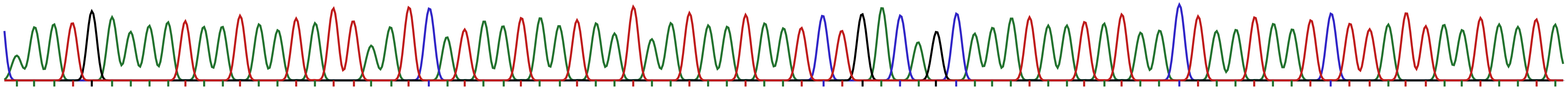

270 280 290 300 310 320 330 340
A A A T G A A A A T A A T A A T A T T A A T C A T A A T A A T A A T A A T A A T A A T A A T C T G A C A G C A A A T A A T A T A A C T A A T A A T C T T A T T A A T A A T A

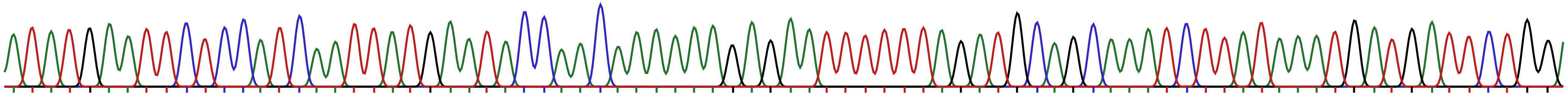

350 360 370 380 390 400 410 420
A T A T G A A T T C T C C A T C A A T T A T G A A T A C C A A C A A A A A A G A G A A T T T T T T A G A T G C A G C A A A T C T T A T A A A T G A T G A T T C T G G

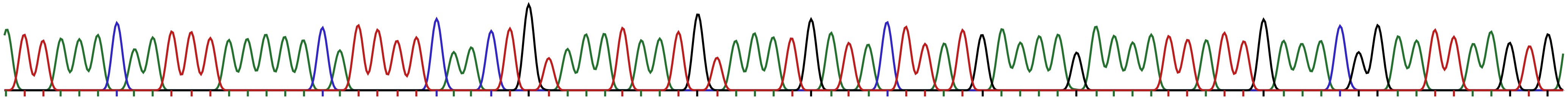

430 440 450 460 470 480 490 500
A T T A A A C A A T T T A A A A A C A T T T T C A A C T G T A A A T A A T G T A A A T G A T A C T T A T G A A A A G A A A A T T A T T G A A A C G G A A T T A A G T G

Samples: 12944
Bases: 1088
Average spacing: 12

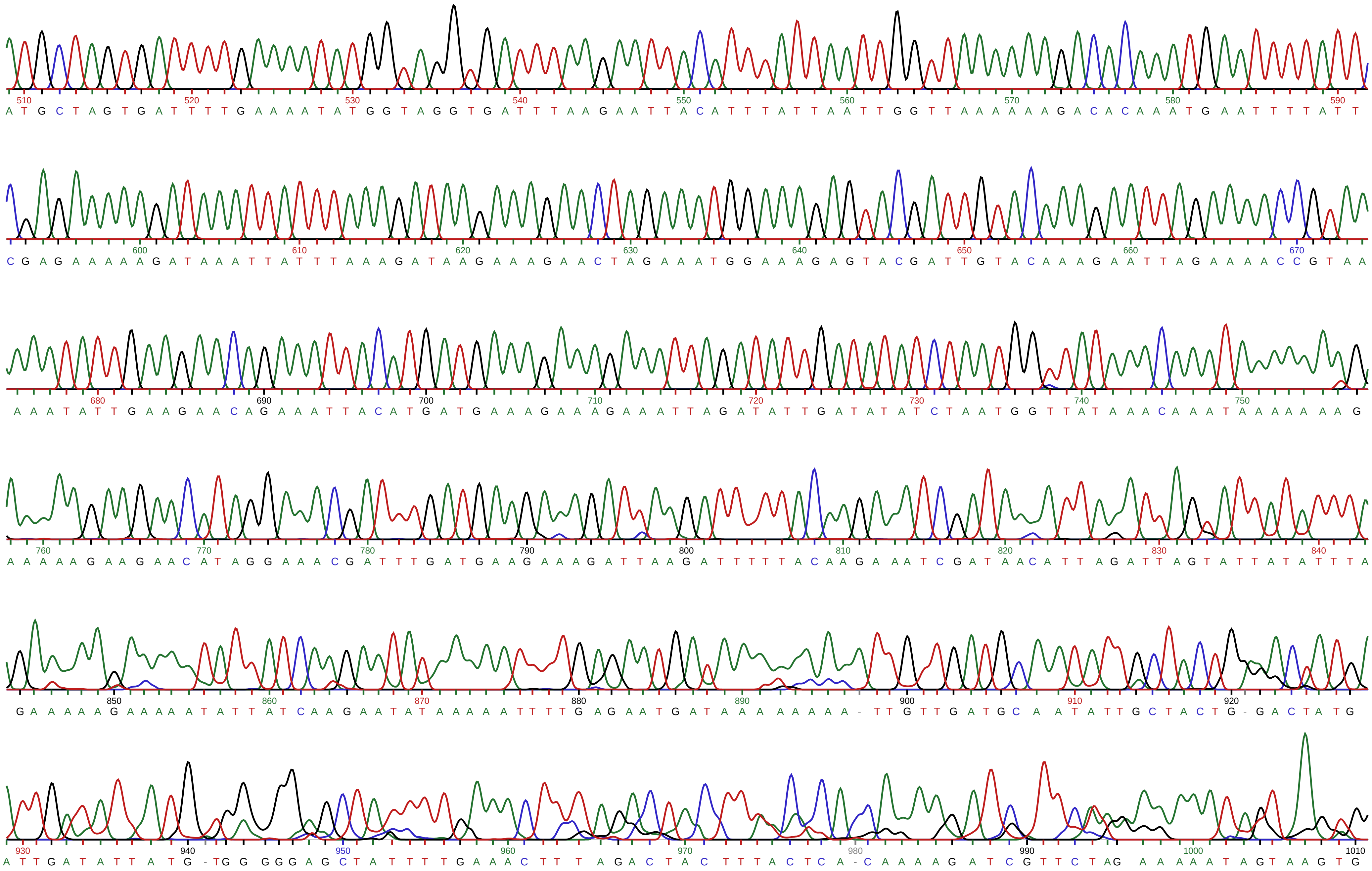

Samples:	12944	
Bases:	1088	
Average spacing:	12	

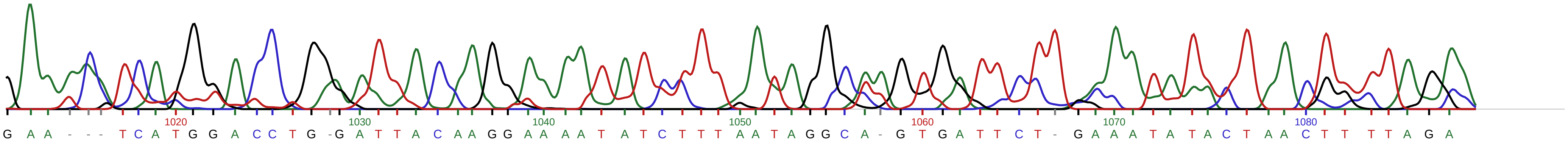

Supplement: Supporting information 2 — (ZIP) [file pone.0316479.s002.zip › 044KN2F_PREMIX_Plate_KELCH2_A01.pdf]
